# Vasoprotective Endothelial Effects of Chronic Cannabidiol Treatment and Its Influence on the Endocannabinoid System in Rats with Primary and Secondary Hypertension

**DOI:** 10.3390/ph14111120

**Published:** 2021-10-31

**Authors:** Marta Baranowska-Kuczko, Hanna Kozłowska, Monika Kloza, Magdalena Kusaczuk, Ewa Harasim-Symbor, Michał Biernacki, Irena Kasacka, Barbara Malinowska

**Affiliations:** 1 Department of Experimental Physiology and Pathophysiology, Medical University of Białystok, ul. Mickiewicza 2A, 15-222 Białystok, Poland; hkozl@umb.edu.pl (H.K.); monika.kloza@umb.edu.pl (M.K.); barbara.malinowska@umb.edu.pl (B.M.); 2 Department of Clinical Pharmacy, Medical University of Białystok, ul. Mickiewicza 2A, 15-222 Białystok, Poland; 3 Department of Pharmaceutical Biochemistry, Medical University of Białystok, ul. Mickiewicza 2A, 15-222 Białystok, Poland; mkusaczuk@wp.pl; 4 Department of Physiology, Medical University of Białystok, ul. Mickiewicza 2C, 15-222 Białystok, Poland; eharasim@umb.edu.pl; 5 Department of Analytical Chemistry, Medical University of Białystok, ul. Mickiewicza 2D, 15-222 Białystok, Poland; michal.biernacki@umb.edu.pl; 6 Department of Histology and Cytophysiology, Medical University of Białystok, ul. Mickiewicza 2C, 15-222 Białystok, Poland; kasacka@umb.edu.pl

**Keywords:** cannabidiol, vascular (endothelial) dysfunction, SHR, DOCA-salt, endocannabinoids, eNOS

## Abstract

Our study aimed to examine the endothelium (vascular)-protecting effects of chronic cannabidiol (CBD) administration (10 mg/kg once daily for 2 weeks) in aortas and small mesenteric (G3) arteries isolated from deoxycorticosterone-induced hypertensive (DOCA-salt) rats and spontaneously hypertensive rats (SHR). CBD reduced hypertrophy and improved the endothelium-dependent vasodilation in response to acetylcholine in the aortas and G3 of DOCA-salt rats and SHR. The enhancement of vasorelaxation was prevented by the inhibition of nitric oxide (NO) with L-NAME and/or the inhibition of cyclooxygenase (COX) with indomethacin in the aortas and G3 of DOCA-salt and SHR, respectively. The mechanism of the CBD-mediated improvement of endothelial function in hypertensive vessels depends on the vessel diameter and may be associated with its NO-, the intermediate-conductance calcium-activated potassium channel- or NO-, COX-, the intermediate and the small-conductance calcium-activated potassium channels-dependent effect in aortas and G3, respectively. CBD increased the vascular expression of the cannabinoid CB_1_ and CB_2_ receptors and aortic levels of endocannabinoids with vasorelaxant properties e.g., anandamide, 2-arachidonoylglycerol and palmitoyl ethanolamide in aortas of DOCA-salt and/or SHR. In conclusion, CBD treatment has vasoprotective effects in hypertensive rats, in a vessel-size- and hypertension-model-independent manner, at least partly via inducing local vascular changes in the endocannabinoid system.

## 1. Introduction

Endothelial (vascular) dysfunction is implicated in many diverse human panvascular diseases, including hypertension. It is a multifactorial process characterized mainly by impaired endothelium-dependent vasodilation due to decreased nitric oxide (NO) synthesis and availability, inflammation, increased reactive oxygen species production, vascular remodeling and acute infections, particularly viral diseases [1,2,3]. Pharmacotherapies improving endothelial function with beneficial pleiotropic effects are therefore considered a central target in the treatment of hypertension and other cardiovascular diseases [2,3,4].

Cannabidiol (CBD) is a non-intoxicating and generally safe and well-tolerated ingredient of cannabis that is already on the market, licensed and approved as Epidiolex^®^ in 2018 by the US Food and Drug Administration for children with severe drug-resistant epilepsy, and internationally approved for the therapy of spasticity in multiple sclerosis. It has also received orphan designation for the treatment of neonatal hypoxia-ischemic encephalopathy [5,6,7,8]. A number of preclinical and clinical studies have also shown it to possess desirable properties, such as antioxidant, anti-inflammatory, immunomodulatory, neuroprotective, procognitive, anti-anxiety, antipsychotic and anti-proliferative effects, which can be used for other clinical conditions, including cardiovascular diseases and hypertension [5,6,7]. The multipharmacological profile of CBD corresponds well with its multidirectional influence on the cardiovascular system, especially under pathophysiological conditions, when the endocannabinoid system, which may play a protective role in hypertension, at least in the vascular system, is overactivated [4,5,9,10,11]. Chronic CBD administration at a dose of 10 mg/kg diminished monocrotaline-induced pulmonary hypertension in rats [12] and exerted antioxidant effects and altered cardiac and plasma endocannabinoid levels in deoxycorticosterone-salt-induced hypertensive rats (DOCA-salt) and spontaneously hypertensive rats (SHR) [13]; however, it did not affect systemic blood pressure (BP) in these animals [12,13]. Acute administration reduced increases in BP induced by stress but not under control conditions [14,15]. Moreover, it decreased diastolic BP in pithed (but not in anaesthetized) SHR and normotensive control Wistar–Kyoto (WKY) animals, confirming the involvement of CBD-induced peripheral vasodilatation in decreasing BP [5].

The vasodilatory effect of CBD is the most consistent effect of this compound in the cardiovascular system and has been demonstrated on isolated human and animal vessels under both physiological [10,16,17,18,19] and pathological [10,20,21] conditions. CBD-mediated relaxation has been demonstrated in human mesenteric [19] and pulmonary [10] arteries and in rat aortas [17] and femoral [18] and mesenteric arteries under normotensive and hypertensive conditions [10]. The vasodilatory effects of CBD in the human mesenteric artery were dependent on the endothelium, the potassium channel, the transient receptor potential vanilloid 1 (TRPV1), cyclooxygenase (COX) derivatives, NO and the cannabinoid CB_1_ receptors; those in the rat aorta depended on peroxisome proliferator-activated receptors (PPARs), calcium channel inhibition and superoxide dismutase (SOD) [17,19]. The protective role of CB_1_ receptors in the vasodilatory effect of CBD in mesenteric arteries has been indicated only in hypertensive rats and not in normotensive control rats [10]. Moreover, CBD augmented endothelium-dependent vasodilatation in response to acetylcholine (Ach) in the mesenteric arteries of Zucker diabetic fatty (ZDF) rats both ex vivo and in vivo via the cannabinoid CB_2_ and prostanoid EP4 receptors, and elevated SOD and COX and nitric oxide synthase (NOS) activity [20,21]; it had a similar effect in the pulmonary arteries of monocrotaline-induced pulmonary hypertensive rats via an unknown mechanism [12]. Very recently, it has been suggested that CBD in the clinical range (1–3 μM) could be therapeutic for peripheral vascular disease, which is characterized by decreased vascular tone induced by many vasoconstrictors in arterial circulation but not in the heart and non-vascular smooth muscle [22].

We took into consideration the following: (1) The vasodilatory activity of CBD and (2) its other vasoprotective properties, such as anti-proliferative [23], antioxidant and anti-inflammatory effects (in hypertension) [13]; its abilities to decrease arterial stiffness, to improve endothelial function [15] and to increase cerebral blood flow during occlusion [24,25]; and its vascular-stabilizing effect in LPS-induced encephalitis [26] and diabetes [27,28]. (3) CBD is an inhibitor of fatty acid amide hydrolase (FAAH), an enzyme responsible for the degradation of anandamide and other endocannabinoids. (4) We have demonstrated beneficial changes in the vascular endocannabinoid systems of rats with primary and secondary hypertension in a vessel-size-dependent manner [4,9]. Therefore, the aim of our study was to determine whether chronic CBD administration had an endothelium (vascular)-protecting effect in conduit vessels and resistant vasculature, i.e., aortas and mesenteric G3 arteries isolated from hypertensive rats (DOCA-salt and SHR) and their normotensive controls, sham-operated (SHAM) and WKY rats, respectively. Moreover, we searched for possible targets for a CBD-mediated vasoprotective mechanism in hypertension, focusing on endothelium- and cannabinoid-related vasomediators, i.e., NO, COX derivatives, intermediate and small calcium-activated potassium channels (K_Ca_3.1 and K_Ca_2.3, respectively), the cannabinoid CB_1_ and CB_2_ and vanilloid TRPV1 receptors, and a wide range of endocannabinoids.

## 2. Results

### 2.1. General

The systolic blood pressure (SBP) before the first dose of CBD (or its vehicle) was higher in DOCA-salt rats and SHR than in age-matched SHAM and WKY rats (142 ± 4 mmHg, *n* = 8, vs. 117 ± 9 mmHg, *n* = 8, *p* < 0.05; 190 ± 6 mmHg, *n* = 8, vs. 109 ± 7 mmHg, *n* = 8, *p* < 0.001) (described previously [13,29]). Chronic CBD administration for two weeks failed to modify SBP in DOCA-salt rats and SHR (169 ± 13 mmHg, *n* = 8; 185 ± 5 mmHg, respectively) and in SHAM and WKY (106 ± 7 mmHg; *n* = 8; 114 ± 5 mmHg; *n* = 8, respectively). The mean tension levels [in mN] induced by phenylephrine in aortas (7.3 ± 0.6, *n* = 25; 7.4 ± 0.8, *n* = 23; 7.5 ± 0.5, *n* = 21; 7.3 ± 0.7, *n* = 22; 7.0 ± 0.5, *n* = 26; 7.4 ± 0.6, *n* = 24; 6.8 ± 0.6, *n* = 27; 6.8 ± 0.6, *n* = 28) and small mesenteric G3 arteries (11.4 ± 2.4, *n* = 23; 11.8 ± 1.5, *n* = 20; 11.5 ± 2.6, *n* = 20; 11.8 ± 2.7, *n* = 23; 12.0 ± 2.9, *n* = 24; 10.9 ± 2.8, *n* = 28; 13.1 ± 2.0, *n* = 23; 11.5 ± 1.8, *n* = 25) were comparable in all the experimental groups (i.e., SHAM, SHAM + CBD, DOCA-salt, DOCA-salt + CBD, WKY, WKY + CBD, SHR and SHR + CBD). No significant effects of L-N^G^-nitroarginine methyl ester (L-NAME) and indomethacin on vessel function were observed.

### 2.2. Influences of Hypertension and Chronic Administration of CBD on Vasodilatory Effects of Acetylcholine and Sodium Nitroprusside in Aortas and Mesenteric G3 Arteries

**Figure 1:**
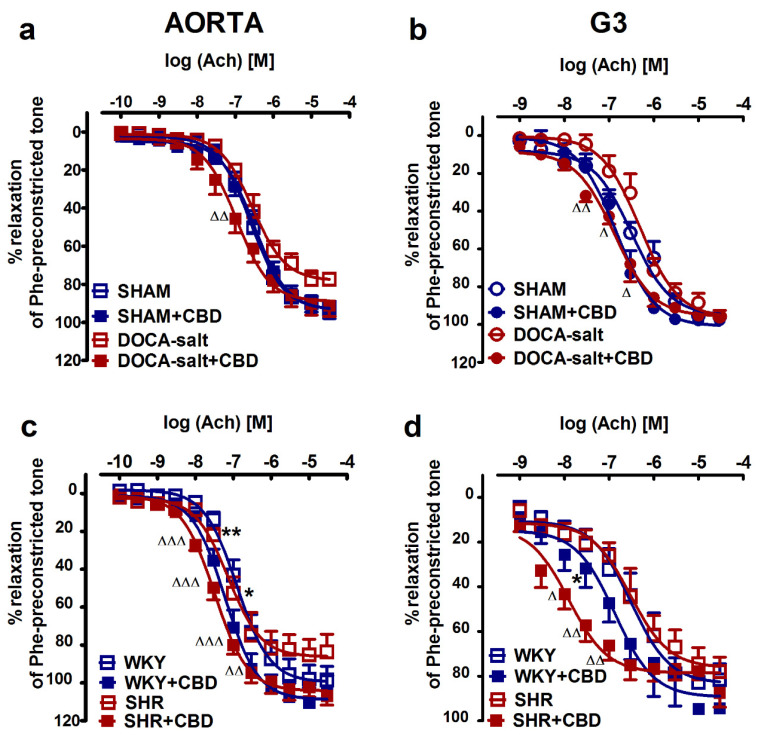
Influences of cannabidiol (CBD) and its vehicle on the vasorelaxant effects of acetylcholine (Ach) in aortas (**a**,**c**) and the mesenteric G3 arteries (**b**,**d**) isolated from deoxycorticosterone-induced (DOCA-salt) and spontaneously (SHR) hypertensive rats and their normotensive controls, sham-operated (SHAM) and Wistar–Kyoto (WKY) rats, respectively. CBD (10 mg/kg) or its vehicle was injected i.p. every 24 h for 14 days. Vasodilatory responses are shown as percentages of the reference response of the isometric contraction induced by phenylephrine (Phe). Mean ± SEM of *n* = 5–8 tissues for each curve. *^,∆^ *p* < 0.05, **^,∆∆^ *p* < 0.01 and ^∆∆∆^ *p* < 0.001 compared to the respective control groups (* SHAM/WKY or ^∆^ DOCA-salt/SHR), as determined by one-way ANOVA, followed by Bonferroni’s multiple-comparison tests. In a few cases, the SEM is smaller than or equal to the size of the symbol. See Table 1 and Table 2 for statistical analysis.

**Figure 2:**
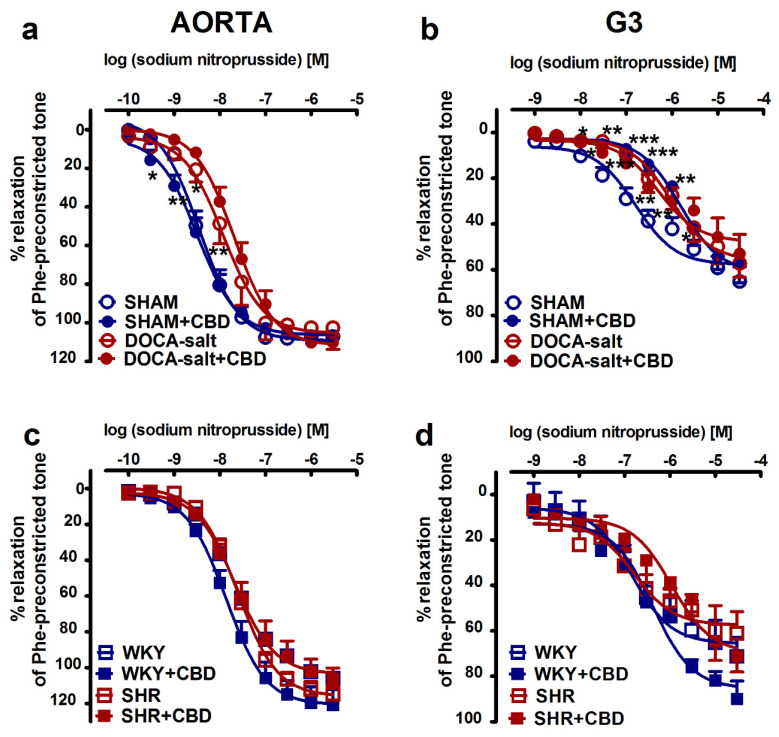
Influences of cannabidiol (CBD) and its vehicle on the vasorelaxant effects of sodium nitroprusside in aortas (**a**,**c**) and the mesenteric G3 arteries (**b**,**d**) isolated from deoxycorticosterone-induced (DOCA-salt) and spontaneously (SHR) hypertensive rats and their normotensive controls, sham-operated (SHAM) and Wistar–Kyoto (WKY) rats, respectively. CBD (10 mg/kg) or its vehicle was injected i.p. every 24 h for 14 days. Vasodilatory responses are shown as percentages of the reference response of the isometric contraction induced by phenylephrine (Phe). Mean ± SEM of *n* = 5–8 tissues for each curve. * *p* < 0.05, ** *p* < 0.01 and *** *p* < 0.001 compared to the respective control groups (* SHAM/WKY), as determined by one-way ANOVA followed by Bonferroni’s multiple-comparison tests. In a few cases, the SEM is smaller than or equal to the size of the symbol. See Table 1 and Table 2 for statistical analysis.

To determine the effects of hypertension and chronic CBD administration on vessel endothelium and muscle function, we examined phenylephrine-preconstricted endothelium-intact rings’ responses to acetylcholine (Ach; aortas: 0.0001–30 µM, and mesenteric G3 arteries: 0.001–30 µM) and sodium nitroprusside (SNP; aortas: 0.0001–3 µM, and mesenteric G3 arteries: 0.001–30 µM), which cause vascular relaxation dependent on the endothelium or NO donor, respectively. In normotensive and hypertensive rats, Ach (Figure 1) and SNP (Figure 2) produced concentration-dependent relaxation of isolated rat aortas (Figure 1a,c and Figure 2a,c) and mesenteric G3 arteries (Figure 1b,d and Figure 2b,d). No significant differences in the potency and efficacy of Ach between the hypertensive groups and their normotensive controls were recorded (Table 1 and Table 2). However, the vasodilatory responses to Ach in aortas tended to decrease by about 15% both in DOCA-salt rats and in SHR. By contrast, the SNP-mediated vasorelaxation in DOCA-salt rats was shifted to the right by factors of 3 and 6 in aortas and mesenteric G3 arteries, respectively. In SHR, the responses to SNP were unchanged in aortas and in mesenteric G3 arteries when compared to WKY. CBD modified endothelium-dependent vasorelaxant responses independently to artery size and etiology of hypertension. It enhanced the potency of Ach-induced vasorelaxation in aortas and in mesenteric G3 arteries by 2- and 4-fold in DOCA-salt rats and in SHR, respectively. Similarly, chronic CBD administration augmented the vasorelaxation response to Ach in normotensive mesenteric G3 arteries of WKY and tended to increase vasorelaxation in the aortas of WKY and mesenteric G3 arteries of SHAM. By contrast, CBD diminished the relaxant potency of SNP in G3 arteries isolated from SHR by about 6.5-fold and from SHAM by 10-fold (for the pEC_50_ and R_max_ values, see Table 1 and Table 2; Figure 1 and Figure 2).

### 2.3. Evaluation of Endothelial Mediators Involved in Vascular Effects of Acetylcholine-Induced Relaxation in Aortas and Mesenteric G3 Arteries

**Figure 3:**
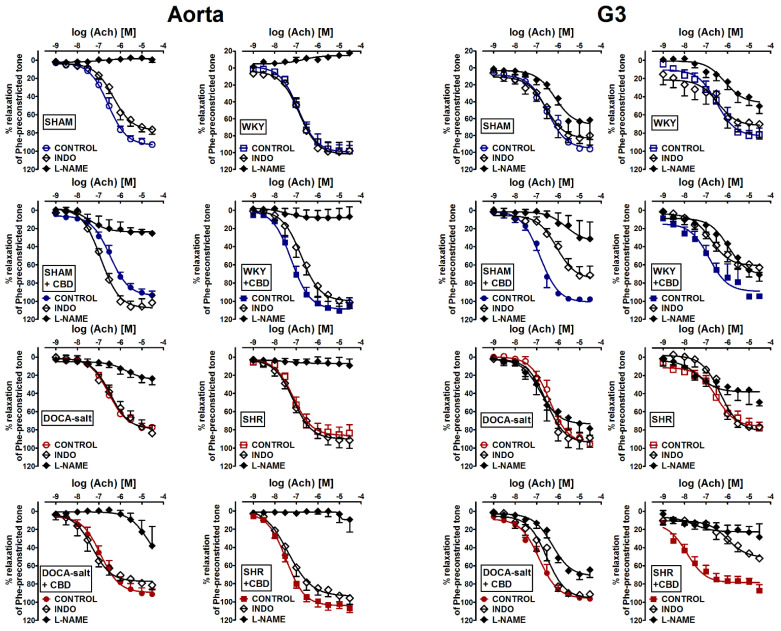
Influence of cannabidiol (CBD) and its vehicle on the vasorelaxant effects of acetylcholine (Ach) in the presence of inhibitors of nitric oxide synthase—N^G^-nitro-L-arginine methyl ester (L-NAME, 300 µM) or cyclooxygenase 1 and 2-indomethacin (INDO, 10 µM) in aortas and the mesenteric G3 arteries isolated from deoxycorticosterone-induced (DOCA-salt) and spontaneously (SHR) hypertensive rats and their normotensive controls, sham-operated (SHAM) and Wistar–Kyoto (WKY) rats, respectively. CBD (10 mg/kg) or its vehicle was injected i.p. every 24 h for 14 days. Vasodilatory responses are shown as percentages of the reference response of the isometric contraction induced by phenylephrine (Phe). Mean ± SEM of *n* = 4–8 tissues for each curve. In a few cases, the SEM is smaller than or equal to the size of the symbols. See Table 1 and Table 2 for statistical analysis.

To determine which endothelial mediators, NO or prostanoid-related compound(s), were involved in the vasorelaxant responses to Ach in aortas and mesenteric G3 arteries isolated from normotensive and hypertensive rats treated or untreated with CBD, endothelium-intact rings were incubated with an inhibitor of endothelial nitric oxide synthase; prostacyclin synthase (eNOS), L-NAME (300 μM), and an inhibitor of COX, indomethacin (10 μM) (Figure 3). The incubation with L-NAME completely abolished the vasodilatory response of Ach in the aortas of SHAM, and reduced it by about 80% in WKY, 70% in DOCA-salt and 90% in SHR. In mesenteric G3 arteries, L-NAME diminished the maximal relaxant effects of Ach in normotensive SHAM rats, WKY rats and SHR by about 40%, and did so by about 20% in DOCA-salt rats. CBD increased, by about 15–20%, the affinity of NO-independent component of the Ach-mediated relaxation in the aortas of SHAM and DOCA-salt rats, and in the mesenteric G3 arteries isolated from WKY rats, since L-NAME caused weaker inhibition in comparison to the respective groups not treated with CBD. However, in mesenteric G3 arteries isolated from SHAM rats, DOCA-salt rats and SHR, CBD tended to increase the NO-dependent relaxation, since L-NAME more strongly reduced the Ach-evoked maximal relaxation in the vascular beds taken from rats treated with CBD than untreated rats (for the pEC_50_ and R_max_, see Table 1 and Table 2, and Figure 3).

Indomethacin (10 μM) decreased or tended to decrease the maximal relaxant effects of Ach by approximately 20% in the aortas and mesenteric G3 arteries of SHAM rats and in the mesenteric G3 arteries of WKY, respectively. It did not change the potency and efficacy of Ach in aortas and mesenteric G3 arteries from hypertensive animals. CBD did not change the maximal response to Ach in the presence of indomethacin in the aortas of normotensive and hypertensive rats from both models; however, it enhanced the Ach potency in SHAM and DOCA-salt rats. In mesenteric G3 arteries of SHAM and WKY rats treated with CBD, the inhibition of COX using indomethacin resulted in the Ach-mediated relaxation being reduced by 30%, and in SHR, it resulted in a reduction of about 40%, whereas in DOCA-salt rats, there was no effect (for the pEC_50_ and R_max_, see Table 1 and Table 2, Figure 3).

The CBD-enhanced Ach-mediated vasorelaxation was abolished or diminished by L-NAME in the aortas of DOCA-salt rats and SHR, and the small mesenteric G3 arteries of DOCA-salt rats, SHR and WKY rats, and by indomethacin in the small mesenteric G3 arteries of DOCA-salt rats and SHR. On the contrary, the potency of the amplificatory effect of CBD on Ach-stimulated relaxation persisted in the presence of indomethacin in the aortas of DOCA-salt rats and SHR (for the pEC_50_ and R_max_ see Table 1 and Table 2; Figure 3). This suggests that CBD enhances Ach-mediated vasorelaxation via an NO-dependent and COX-dependent mechanism in the mesenteric G3 arteries of DOCA-salt rats, SHR and WKY rats, and in aortas, the NO-dependent pathway plays a crucial role in the vasorelaxation.

### 2.4. Influences of Hypertension and Chronic Administration of CBD on Vascular Remodeling and Immunohistochemical Staining of von Willebrand Factor; eNOS, CB_1_ and TRPV1 Receptors in Isolated Aortas and Mesenteric G3 Arteries

**Figure 4:**
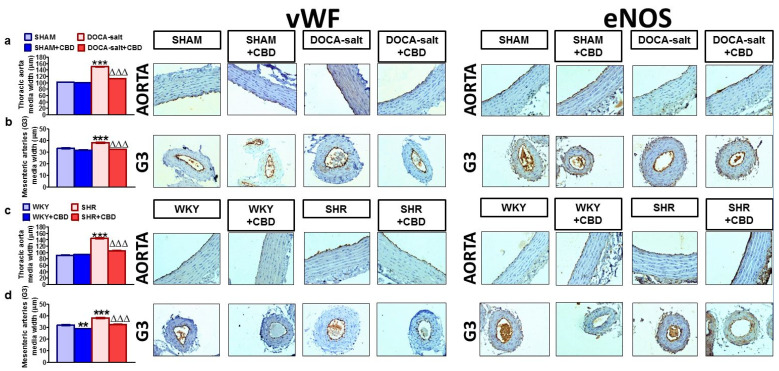
Representative micrographs of the vascular remodeling and influence of cannabidiol (CBD) and its vehicle on immunohistochemical staining of von Willebrand factor (vWF) and endothelial nitric oxide synthase; prostacyclin synthase (eNOS) in cross-sections of the aortic wall and mesenteric G3 arteries isolated from deoxycorticosterone-induced (DOCA-salt) and spontaneously (SHR) hypertensive rats and their normotensive controls, sham-operated (SHAM) and Wistar–Kyoto (WKY) rats, respectively; (magnification 200×). The bar graphs (**a**–**d**) illustrate the measured aortic and mesenteric G3 artery medial widths. H + E staining. CBD (10 mg/kg) or its vehicle was injected i.p. every 24 h for 14 days. Mean ± SEM of *n* = 6 tissues for each bar. ** *p* < 0.01 and ***^,∆∆∆^ *p* < 0.001 compared to the respective control groups (* SHAM/WKY or ^∆^ DOCA-salt/SHR), as determined by one-way ANOVA followed by Bonferroni’s multiple-comparison tests.

**Figure 5:**
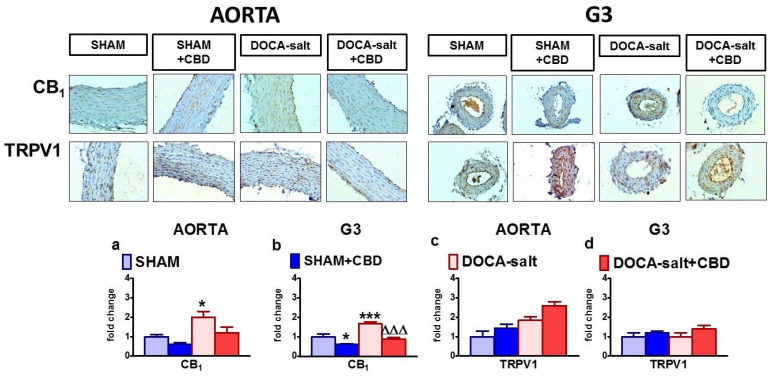
Representative micrographs of the influences of cannabidiol (CBD) and its vehicle on immunohistochemical staining of CB_1_ and TRPV1 receptors in the aortic walls and mesenteric G3 arteries isolated from deoxycorticosterone-induced (DOCA-salt) and normotensive control sham-operated (SHAM) rats (magnification 200×). The bar graphs illustrate the fold changes of the percentage area stained for CB_1_ (**a**,**b**) and for TRPV1 receptors (**c**,**d**) in the aortic walls (**a**,**c**) and mesenteric G3 arteries (**b**,**d**) isolated from deoxycorticosterone-induced (DOCA-salt) and normotensive control sham-operated (SHAM) rats. CBD (10 mg/kg) or its vehicle was injected i.p. every 24 h for 14 days. Mean ± SEM, *n* = 5 tissues for each bar. * *p* < 0.05 and ***^,∆∆∆^ *p* < 0.001 compared to the respective control groups (* SHAM or ^∆^ DOCA-salt), as determined by one-way ANOVA followed by Bonferroni’s multiple-comparison tests.

**Figure 6:**
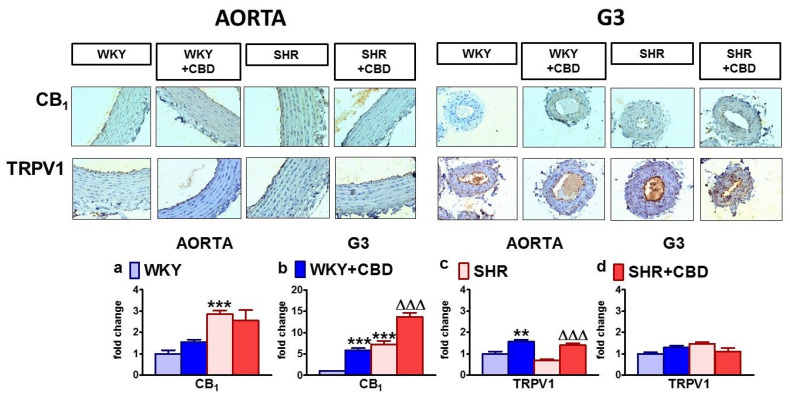
Representative micrographs of influence of cannabidiol (CBD) and its vehicle on immunohistochemical staining of CB_1_ and TRPV1 receptors in the aortic walls and mesenteric G3 arteries isolated from spontaneously (SHR) hypertensive rats and normotensive control Wistar–Kyoto (WKY) rats (magnification 200×). The bar graphs illustrate the fold changes of the percentage area stained for CB_1_ (**a**,**b**) and for TRPV1 receptors (**c**,**d**) in the aortic walls (**a**,**c**) and mesenteric G3 arteries (**b**,**d**) isolated from spontaneously (SHR) hypertensive rats and normotensive control Wistar–Kyoto (WKY) rats. CBD (10 mg/kg) or its vehicle was injected i.p. every 24 h for 14 days. Mean ± SEM, *n* = 5 tissues for each bar. ** *p* < 0.01 and ***^,∆∆∆^ *p* < 0.001 compared to the respective control groups (* WKY or ^∆^ SHR), as determined by one-way ANOVA followed by Bonferroni’s multiple-comparison tests.

Representative images of the vascular remodeling and immunohistochemical staining of various receptors, enzymes and vWF in cross-sections of aortas and mesenteric G3 arteries are shown in Figure 4, Figure 5 and Figure 6. The dark brown precipitate represented the intensity of vWF, eNOS, CB_1_ and TRPV1.

Secondary and primary hypertension induced medial hypertrophy by approximately 50% and 15% in aortas and mesenteric G3 arteries, respectively. CBD reduced the hypertrophy by about 30% in aortas and about 15% in mesenteric G3 arteries from DOCA-salt rats and SHR. It also exhibited anti-proliferative properties in the mesenteric G3 arteries of WKY rats (Figure 4a–d).

The immunostaining for vWF and eNOS revealed their presence in the endothelial cells of the walls of the aortas and mesenteric G3 arteries in all the groups. More intense staining of vWF was observed in the endothelia of both vessels in DOCA-salt rats and SHR, which could confirm impaired endothelial function, as vWF is a marker of endothelial dysfunction [30,31]. The eNOS immunoreactivity was less intense in DOCA-salt rats and showed no changes in the aortas and mesenteric G3 arteries of SHR (Figure 4).

Immunostaining for the CB_1_ and TRPV1 receptors showed that they were present in the endothelial cells and smooth muscle cells of the aortas and mesenteric G3 arteries of SHAM and DOCA-salt rats. The highest intensity of CB_1_ receptor staining was observed in the mesenteric G3 arteries and aortas of DOCA-salt rats (Figure 5).

In the aortas of WKY rats, CB_1_ and TRPV1 receptor staining was mainly observed in endothelial cells. In the mesenteric G3 arteries of WKY rats, the CB_1_ staining was very weak, and TRPV1 receptor expression was observed in both endothelial and smooth muscle cells. In SHR, CB_1_ immunostaining showed elevated levels in both the endothelial and smooth muscle cells of aortas and mesenteric G3 arteries. The TRPV1 receptor staining tended to be weaker in the endothelium of the SHR aortas. (Figure 6).

Chronic CBD treatment decreased and increased the intensity of vWF and eNOS staining, respectively, in aortas and mesenteric G3 arteries in the normotensive and hypertensive groups for both models of hypertension (Figure 4). 

Moreover, CBD enhanced the staining of the CB_1_ receptor in the mesenteric G3 arteries of WKY rats and SHR and attenuated it in SHAM and DOCA-salt rats. In addition, it increased TRPV1 immunostaining in the aortas of WKY rats and SHR. In other cases, CBD failed to modify the immunostaining of the examined proteins (Figure 5 and Figure 6).

### 2.5. Influences of Hypertension and Chronic Administration of CBD on Vascular Expression of CB_1_, CB_2_ and TRPV1 Receptors; COX-1; COX-2; and eNOS in Isolated Aortas and Mesenteric G3 Arteries

**Figure 7:**
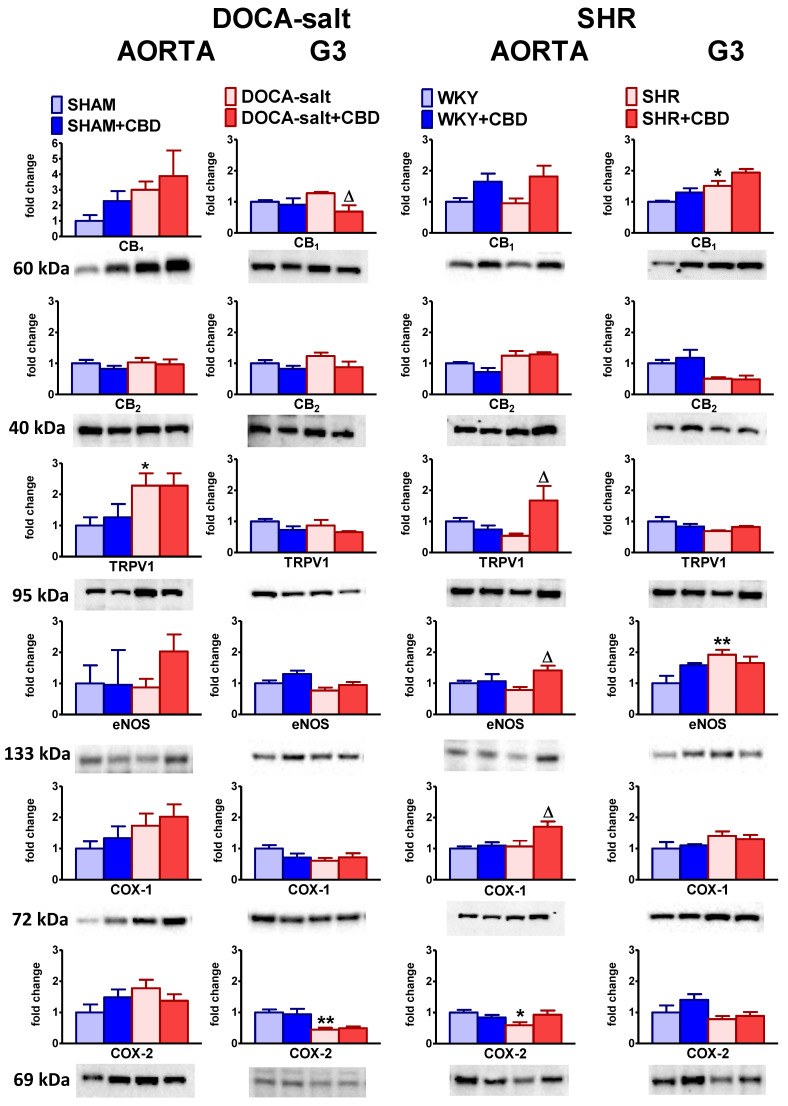
Influence of cannabidiol (CBD) and its vehicle on the expression of CB_1_, CB_2_, TRPV1 receptor, endothelial nitric oxide synthase (eNOS), cyclooxygenases 1 and 2 (COX-1, COX-2) in thoracic aortas and mesenteric G3 arteries isolated from deoxycorticosterone-induced (DOCA-salt) and spontaneously (SHR) hypertensive rats and their normotensive controls, sham-operated (SHAM) and Wistar–Kyoto (WKY) rats, respectively. CBD (10 mg/kg) or its vehicle was injected i.p. every 24 h for 14 days. Vessels were prepared 24 h after the final injection. Receptor protein was determined by Western blots and is given as fraction of the value in the normotensive control (first of the four columns). Images obtained using stain-free gel technology that allows for total protein visualization and quantification are shown with a loading control (Loading). Mean ± SEM, *n* = 5–6 tissues for each bar. *^,∆^ *p* < 0.05 and ** *p* < 0.01 compared to the respective control groups (* SHAM/WKY or ^∆^ DOCA-salt/SHR), as determined by one-way ANOVA followed by Bonferroni’s multiple-comparison tests.

The expression of CB_1_, CB_2_ and TRPV1 receptors; eNOS; COX-1; and COX-2 in isolated aortas and mesenteric G3 arteries was analyzed by Western blotting. It showed separate immunoreactive bands of the molecular sizes expected for CB_1_ receptors (60 kDa), CB_2_ receptors (40 kDa), TRPV1 receptors (95 kDa), eNOS (133 kDa), COX-1 (72 kDa) and COX-2 (69 kDa) for both vessels (*n* = 5–6). As shown in Figure 7, the expression of CB_1_ receptors increased in the mesenteric G3 arteries of SHR and tended to increase in aortas and mesenteric G3 arteries from DOCA-salt rats. Only in the aortas of SHR was the CB_1_ receptor’s expression similar to that of WKY. The CB_2_ receptor’s expression showed a tendency to increase in both the vessels of DOCA-salt rats and the aortas of SHR, and to decrease in the mesenteric G3 arteries of SHR. TRPV1, COX-1 and COX-2 expressions was enhanced or tended to be enhanced in aortas of DOCA-salt, whereas in aortas of SHR they were decreased (COX-2), tended to decrease (TRPV1) or was unchanged (COX-1). eNOS expression was unchanged in aortas of both hypertension models when compared to controls. In small diameter arteries of DOCA-salt COX-2 and eNOS density decreased or tended to decrease, while in SHR eNOS density was augmented and COX-2 density showed a tendency to decline.

Chronic CBD treatment decreased CB_1_ expression in the mesenteric G3 arteries of DOCA-salt rats; increased TRPV1, COX-1 and eNOS expression in the aortas of SHR; and tended to increase the expression of eNOS in the aortas of DOCA-salt rats and CB_1_ expression in the aortas and mesenteric G3 arteries of SHR. We did not observe any significant changes in the expression levels of cannabinoid-related proteins in isolated aortas and mesenteric G3 arteries subjected to CBD in the normotensive controls for both models of hypertension (Figure 7).

### 2.6. Influences of Hypertension and Chronic Administration of CBD on Cnr1, Cnr2, eNOS, PGIS, K_Ca_3.1 and K_Ca_2.3 Gene Expression in Isolated Aortas and Mesenteric G3 Arteries

**Figure 8:**
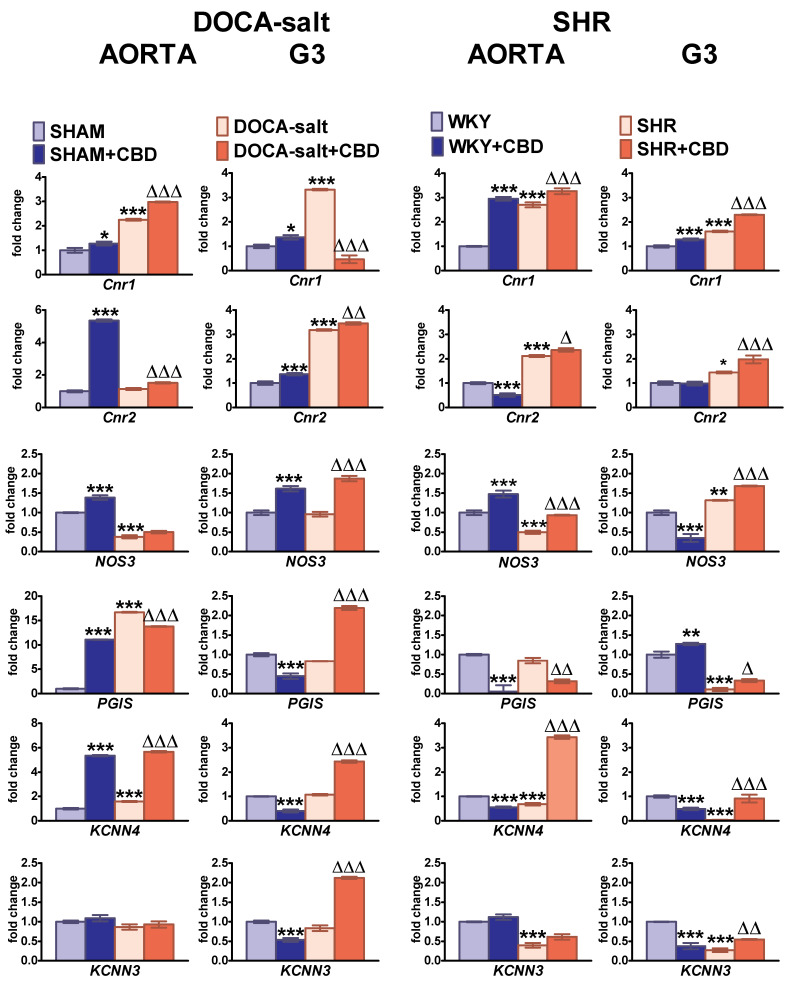
Influence of cannabidiol (CBD) and its vehicle on the relative expression of *Cnr1, Cnr2, NOS3, PGIS, KCNN4* and *KCNN3* as evaluated by real-time quantitative PCR in thoracic aorta and mesenteric G3 arteries isolated from deoxycorticosterone-induced (DOCA-salt) and spontaneously (SHR) hypertensive rats and their normotensive controls, sham-operated (SHAM) and Wistar–Kyoto (WKY) rats, respectively. CBD (10 mg/kg) or its vehicle was injected i.p. every 24 h for 14 days. Results are shown as mean ± SEM (*n* = 6 per group), for the relative fold change in mRNA expression in comparison to the respective control, whose expression level was set to 1. *^,∆^ *p* < 0.05, **^,∆∆^ *p* < 0.01 and ***^,∆∆∆^ *p* < 0.001 compared to the respective control groups (* SHAM/WKY or ^∆^ DOCA-salt/SHR), as determined by one-way ANOVA followed by Bonferroni’s multiple-comparison tests.

The expression of the *Cnr1, Cnr2, NOS3, PGIS, KCNN4* and *KCNN3* genes (i.e., genes for the cannabinoid CB_1_ and CB_2_ receptors; endothelial nitric oxide synthase; prostacyclin synthase; endothelial intermediate (K_Ca_3.1); and small (K_Ca_2.3) conductance calcium-activated potassium channels, respectively) were analyzed by real-time qPCR (Figure 8). *Cnr1* and *Cnr2* expression was upregulated in both types of arteries and hypertension models, in comparison to their respective controls. The one exception was no change in mRNA expression for *Cnr2* in the aortas of DOCA-salt rats. The expression of *NOS3* was downregulated in aortas from DOCA-salt rats and SHR, and in mesenteric G3 arteries, it was not changed and increased in DOCA-salt rats and SHR, respectively. *PGIS* expression increased in the aortas of DOCA-salt rats. It was strongly diminished in the mesenteric resistance vessels of SHR, but it was unchanged in the mesenteric G3 arteries of DOCA-salt rats and the aortas of SHR. Both *KCNN4* and *KCNN3* were downregulated in the aortas and mesenteric G3 arteries of SHR. In DOCA-salt rats, their expression levels were not modified, with one exception—an increase in *KCNN4* expression in aortas.

Chronic CBD treatment enhanced the mRNA expression of almost all the examined genes in both types of vessels isolated from both hypertensive strains. There were only three exceptions: the expression of *Cnr1* in the mesenteric G3 arteries of DOCA-salt rats and *PGIS* expression in the aortas of SHR and DOCA-salt rats were reduced by almost 4-fold and by about 60 and 20%, respectively (Figure 8). In normotensive controls, the chronic administration of CBD upregulated the expression of *Cnr1* in the arteries of SHAM and WKY rats. Additionally, the chronic administration of CBD increased the expression levels of *Cnr2, NOS3*, *PGIS* and *KCNN4* in the aortas of SHAM; *Cnr2* and *NOS3* in the mesenteric G3 arteries of SHAM; and *NOS3* in the aortas and *PGIS* in the mesenteric G3 arteries of WKY. However, it decreased the mRNA of *Cnr2, PGIS* and *KCNN4* in the aortas and *NOS3*, *KCNN4* and *KCNN3* in the mesenteric G3 arteries of WKY (Figure 8).

### 2.7. Influences of Hypertension and Chronic Administration of CBD on Endocannabinoid Levels in Isolated Aortas

**Figure 9:**
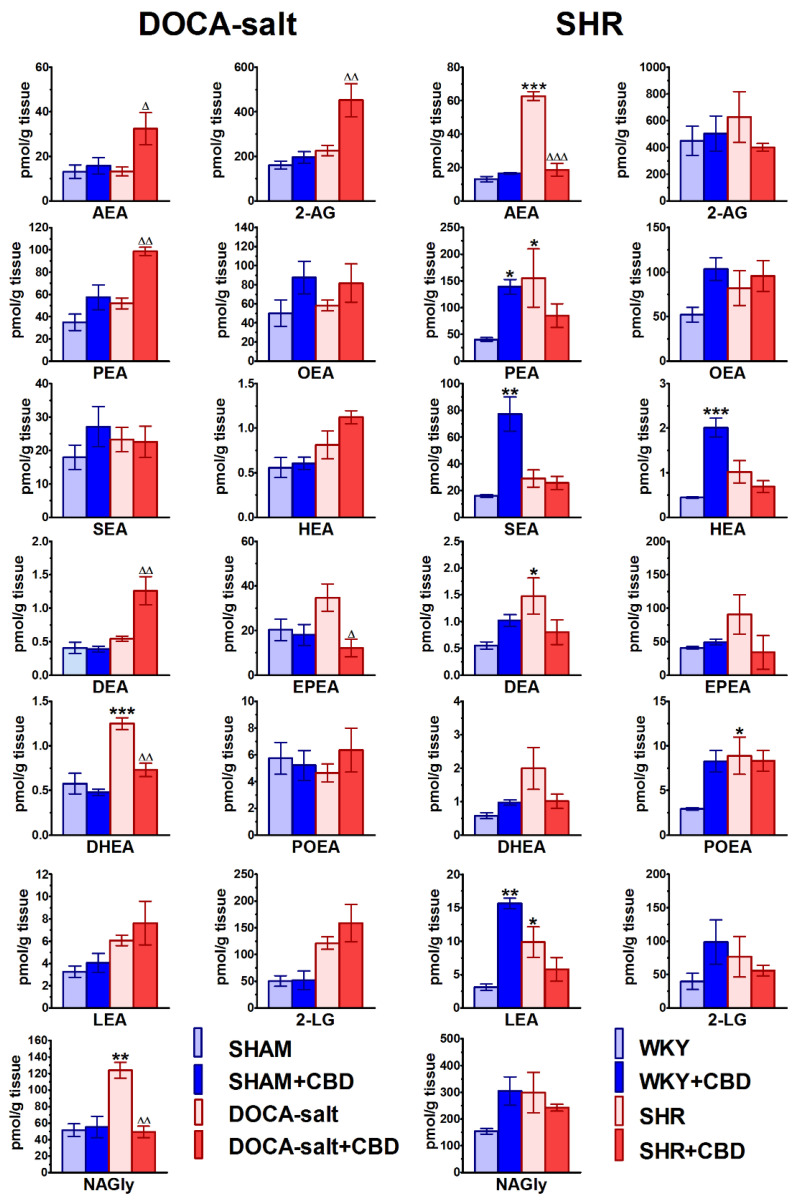
Influences of cannabidiol (CBD) and its vehicle on endocannabinoid levels in thoracic aorta isolated from deoxycorticosterone-induced (DOCA-salt) and spontaneously (SHR) hypertensive rats and their normotensive controls, sham-operated (SHAM) and Wistar–Kyoto (WKY) rats, respectively. CBD (10 mg/kg) or its vehicle was injected i.p. every 24 h for 14 days. Mean ± SEM of *n* = 6 tissues for each bar. *^,∆^ *p* < 0.05, **^,∆∆^ *p* < 0.01 and ***^,∆∆∆^ *p* < 0.001 compared to the respective control groups (* SHAM/WKY or ^∆^ DOCA-salt/SHR), as determined by one-way ANOVA followed by Bonferroni’s multiple-comparison tests. AEA—anandamide; 2-AG—2-arachidonoylglycerol; PEA—palmitoyl ethanolamide; OEA—oleoyl ethanolamide; SEA—stearoyl ethanolamide; HEA—homolinolenyl ethanolamide; DEA—docosatetraenoyl ethanolamide; EPEA—eicosapentaenoyl ethanolamide; DHEA—docosahexaenoyl ethanolamide; POEA—palmitoleoyl ethanolamide; LEA—linolenoyl ethanolamide; 2-LG—linoleoylglycerol; NAGly—*N*-arachidonoyl glycine.

As shown in Figure 9, 2-arachidonoylglycerol (2-AG) showed the highest aortic level among the endocannabinoids. Its contents were 160 and 450 pmol/g (*n* = 6) in the aortas of normotensive SHAM and WKY rats, respectively. The levels of the best-known endocannabinoid anandamide (AEA) in SHAM and WKY rats were about 10 and 35 times lower than the respective values of 2-AG. Although the levels of palmitoyl ethanolamide (PEA), oleoyl ethanolamide (OEA), eicosapentaenoyl ethanolamide (EPEA), 2-linoleoylglycerol (2-LG) and *N*-arachidonoyl glycine (NAGly) were higher than those of AEA, they were still lower than the 2-AG levels in SHAM rats by factors of about 4.5, 3, 8, 3 and 3. In WKY rats, the levels were lower by about 10-fold in the case of PEA, OEA, EPEA and 2-LG, and 3-fold for NAGly. Homolinolenyl ethanolamide (HEA), docosatetraenoyl ethanolamide (DEA), docosahexaenoyl ethanolamide (DHEA), palmitoleoyl ethanolamide (POEA) and linolenoyl ethanolamide (LEA) showed the lowest levels, i.e., about 0.4–5.7 pmol/g tissue. Interestingly, the aortic levels of almost all the endocannabinoids were similar in SHAM and WKY rats, with the exceptions of EPEA and NAGly, which were two and three times higher in WKY than in SHAM, respectively, and POEA (two times lower in WKY).

Hypertension elevated or tended to elevate the aortic levels all of the endocannabinoids in SHR. In DOCA-salt rats, the levels of AEA, 2-AG, OEA, DEA and EPEA were not changed; the levels of DHEA and NAGly were increased; and the levels of PEA, SEA, HEA, EPEA, LEA and 2-LG slightly increased (Figure 9). The most significant increases were observed for AEA and PEA, whose contents in SHR were about five and four times higher, respectively, than in WKY rats. The DEA, POEA and LEA levels increased by about three times in comparison to those in WKY rats. Most of the remaining endocannabinoid levels in SHR and DOCA-salt rats were increased by about two-fold.

Chronic CBD administration modified vascular endocannabinoid levels in a model- and BP-dependent manner (Figure 9). Thus, in DOCA-salt rats, it increased the contents of AEA, 2-AG, PEA and DEA by factors of 2–2.5 and tended to enhance the levels of OEA, HEA, POEA, LEA and LG. By contrast, in SHR, it decreased the AEA content by about 70% and tended to reduce the levels of 2-AG, PEA, HEA, DEA, EPEA, DHEA, LEA, 2-LG and NAGly. Moreover, CBD reduced the EPEA, DHEA and NAGly levels in DOCA-salt rats by about 70%, 50% and 60%, respectively. Under normotension, chronic CBD administration increased the contents of aortic SEA, HEA and LEA by a factor of five, and PEA by 3.5-fold in WKY rats, but did not modify the aortic endocannabinoid levels in SHAM rats.

## 3. Discussion

Hypertension is one of the most important and preventable risk factors for cardiovascular morbidity and mortality. It is characterized by a vascular phenotype of endothelial dysfunction, structural remodeling, vascular inflammation and increased stiffness [1,2,3]. Thus, the present study investigated the effects of the chronic administration of CBD on an endothelial (vascular) dysfunction and local vascular endocannabinoid system in experimental hypertension models.

We used two hypertension models with different etiologies, but both were associated with vascular target-organ damage, including vascular dysfunction and remodeling [9,32]: (1) genetic SHR resembling the hypertensive phenotypes of human hypertension, used for the screening of antihypertensive agents [32]; (2) DOCA-salt-induced hypertension with some features of human low-renin hypertension. It is an appropriate model for evaluating the role of sodium, one of the main factors leading to severe hypertension [32].

CBD was given i.p. once daily for 14 days at a dose of 10 mg/kg. The dose of 10 mg/kg diminished the BP and heart rate (HR) in anaesthetized SHR and WKY rats [5] and the stress-induced elevation in BP and HR in conscious rats [33] and humans (i.e., ~600 mg/70 kg) [14,15]. Moreover, its chronic administration improved the endothelium-dependent vasorelaxation in the mesenteric arteries of diabetic rats [21], ameliorated rat pulmonary hypertension [12], reduced the carbachol-induced increases in coronary perfusion pressure in the hearts of hypertensive DOCA-salt rats and SHR [29], and reduced the cardiac and plasma oxidative stress in these animals [13]. It also showed cardioprotective effects in mouse cardiomyopathy induced by doxorubicin [34] and by diabetes [35].

Clinically, endothelial function and dysfunction are mainly evaluated through assessments of endothelium-dependent relaxation, for example, in response to Ach [1,2,3]. The most used approach for directly evaluating vascular function is an organ bath and/or myography technique [32,36], with better access to the vascular endothelium achievable with wire myography [36]. We examined conduit (aortas) and resistance vessel (mesenteric G3 arteries) because hypertension and cannabinoids induce vascular changes depending on the vessel size (for relevant literature, see the Introduction).

### 3.1. Vascular Changes Related to Hypertension

The typical vascular changes related to hypertension, such as a vascular phenotype (endothelial dysfunction), arterial remodeling, vascular inflammation and increased stiffness, were confirmed in other studies (reviewed, for example, by [32,37]). Thus, both in aortas and/or in mesenteric G3 arteries of DOCA-salt rats and SHR, we noticed significant wall hypertrophy with thickening of the vascular media and higher immunoreactivity for vWF. The von Willebrand factor is produced exclusively by endothelial cells. It is a marker of their activation and endotheliopathy connected with inflammation-mediated vascular damage elicited by high systemic BP [31].

We confirmed our previous observations regarding vascular function and the mechanism(s) responsible for vasodilator responses [4,9]. Thus, the endothelium-independent vasorelaxation in response to SNP (its potency) was impaired in the aortas and mesenteric G3 arteries of DOCA-salt rats but not SHR. By contrast, only a tendency to decrease the maximal effect of endothelium-dependent Ach-induced vasodilatation was noticed in aortas from DOCA-salt rats and SHR compared with SHAM and WKY rats. These observations are with accordance with previous studies [4,9] and suggest that the aorta is more vulnerable to this pathological condition than mesenteric G3 arteries, and that the conduit artery rather than the resistance artery is better for measuring endothelium-dependent relaxation as an early indicator for the progression of vascular diseases [38]. The endothelial function may be reduced, unchanged or augmented, depending on the age, artery type and methods used for the determination of vascular function [4,39]. Patients with mild essential hypertension were found to undergo small vessel remodeling, whereas only 60% had endothelial dysfunction [32].

The contribution of NO in endothelium-mediated relaxation varies between vascular beds. It was mainly mediated by NO in the aortas because L-NAME almost abolished the Ach-stimulated dilatation, and in DOCA-salt rats, NO-dependent relaxation slightly decreased when compared to that in SHAM rats. In the mesenteric G3 artery, a greater (about 20%) significance of NO-mediated vasorelaxation in WKY rats and SHR than in DOCA-salt rats was determined. Like in our previous studies, vasodilator prostanoids play a small but constant role in modulating vascular tone independent of the vessel size, especially in normotensive rats [40,41].

The mechanistic changes in the functional studies correlated with changes in the vascular expression of *NOS3* and eNOS. Thus, in the aortas of DOCA-salt rats and SHR, the expression of NOS3 was reduced without significant changes in its protein expression. In small mesenteric G3 arteries, the expression of genes/proteins of the NO-dependent pathway varied according to the hypertension etiology. In primary hypertension, NO-induced vasorelaxation seems to have greater significance, as the expression of both *NOS3* and eNOS increased. In the resistance arteries of DOCA-salt rats, there was no change in *NOS3* or eNOS expression. In contrast to the clear modification of NO-dependent relaxation in hypertension, we did not observe any evident changes in COX-dependent effects regarding functional responses or in biochemical parameters.

Additionally, we evaluated the expression of the mRNAs of *KCNN4* and *KCNN3*, whose products (the intermediate and small calcium-activated potassium channels K_Ca_3.1 and K_Ca_2.3, respectively) are thought to mediate endothelium-dependent hyperpolarization (EDH). They play a pivotal role in NO-/PGI_2_-independent endothelium-mediated vasorelaxation in resistance vessels and act as a backup system to maintain endothelial function in situations associated with decreased bioactivity of NO—e.g., in hypertension [40]. We revealed changes dependent on the hypertension model, since both *KCNN4* and *KCNN3* were downregulated in the aortas and mesenteric G3 arteries of SHR, whereas their expression levels were unchanged in DOCA-salt rats, with the exception of the *KCNN4* level being enhanced in aortas. A similar model of vasomediator expression levels being dependent on the artery size and hypertension model was previously described [40].

The first simultaneous determination of the levels of 13 endocannabinoids and endocannabinoid-related lipids in the aortas of two models of hypertensive rats allowed us to determine the dependence on the hypertension model. The levels of endocannabinoids and endocannabinoid-like compounds mainly increased or tended to increase in the aortas of SHR, whereas in DOCA-salt rats, they were unchanged in comparison to SHAM—only DHEA and NAGly increased. Interestingly, the opposite changes were determined in hearts; the same compounds decreased in SHR and increased in DOCA-salt rats [13]. Most of the ligands measured are FAAH-sensitive. However, no change in FAAH activity in the aortas of DOCA-salt rats [9]—or any tendency toward an increase in SHR [4]—was determined. Therefore, another mechanism could have been responsible for the latter effects—e.g., endocannabinoid synthesis is favored in hypertension [11] and/or AEA transporter activity was decreased [42].

As in the hearts of hypertensive and normotensive rats [13], in aortas, 2-AG had the highest concentrations. The order of the other endocannabinoids by concentration was NAGly > 2-LG ≈ PEA ≈ OEA ≈ EPEA > AEA ≈ SEA > POEA ≈ LEA > HEA ≈ DEA ≈ DHEA. Importantly, the concentrations of NAGly and 2-LG were higher in aortas than in lungs [12]. NAGly is known for its role in endothelium-dependent vasodilation [43,44] and might potentiate carbachol-induced vasorelaxation [43]. 2-LG is described as a partial agonist of the human CB_1_ receptor [45].

Other endocannabinoids diminish vascular tone directly or indirectly through “entourage” action (AEA [11]; 2-AG [46]; PEA and OEA [47]; OEA [48]; reviewed by [6,44]). They can also be vasoprotective ascribed to the CB_2_, CB_1_ and TRPV1 receptors against changes induced by elevated BP (reviewed by [11,44,49]). PEA reduces intraocular pressure in patients with glaucoma and ocular hypertension [50], causes hypotension [51] and attenuates inflammation and oxidative stress resulting from vascular injuries [52].

We confirmed the presence and localization of the CB_1_, CB_2_ and TRPV1 receptors in the endothelium and smooth muscle cells in the aortas and mesenteric G3 arteries of DOCA-salt rats, SHR and their controls. The exception was TRPV1 immunoreactivity, which was observed only in the endothelial cells of aortas isolated from SHAM and DOCA-salt rats, and their vascular density increased in the aortas of DOCA-salt rats only. The expression of the CB_1_ receptor gene and its protein level were increased or somewhat increased in two hypertensive models and both vascular tissues. The *Cnr2* level was unchanged in the aortas of DOCA-salt rats but enhanced in other cases. However, the expression of the CB_2_ receptor protein was unchanged. A similarly increased expression of the CB_1_ and CB_2_ receptors in mesenteric G3 arteries was observed, in contrast to there being no changes in the aortas of DOCA-salt rats and SHR [9,4, respectively].

In summary, our results suggest that the expression levels of enzymes/factors responsible for the syntheses/mechanisms of action of vasodilatory mediators vary among secondary and primary hypertension and arteries of different sizes. Fluctuations in endocannabinoid system expression could be responsible for maintained endothelium-dependent vasorelaxation, despite the medial hypertrophy and endotheliopathy occurring in the vasculature of DOCA-salt rats and SHR. Importantly, we have determined two main beneficial properties of the vascular endocannabinoid system in hypertension: (1) The upregulation of the vascular cannabinoid CB_1_ receptor in DOCA-salt rats and SHR. The CB_1_ receptor activated by endocannabinoids may play a local (in vasculature) protective role against hypertension development via direct vasodilation, indirectly via local feedback diminishing the increases in contraction elicited by various factors [4,9], or via mediating the presynaptic inhibition of the noradrenaline release from the sympathetic nerve endings innervating the resistance vessels of pithed rats [11]. (2) Increased levels of vasodilatory endocannabinoids in SHR.

### 3.2. Vascular Changes Induced by CBD in Hypertension and Normotension

Our present study revealed that chronic CBD treatment exerted three main beneficial vascular effects in both hypertensive models. Firstly, chronic CBD decreased the structural remodeling of the vascular walls of the large and mesenteric G3 arteries in both hypertension models, i.e., it reduced the lumen diameter and thickening of the vascular media, therefore reducing risk factors for end target-organ effects. Moreover, the inhibition of vascular remodeling may have alleviated endothelial dysfunction and reduced the expression of vWF immunostaining upon CBD treatment. Previously, CBD at 10 mg/kg for 2 weeks diminished the widths of the left-ventricle cardiomyocytes of DOCA-salt rats and SHR [29], probably resulting from its anti-inflammatory and antioxidative properties [13]. Moreover, its in vitro application reduced the proliferation of human umbilical artery smooth muscle cells [23].

Secondly, CBD enhanced the vasodilatory effect of Ach in aortas and mesenteric G3 arteries isolated from both strains of hypertensive rats, but not in those from normotensive rats. The unique protective properties of CBD in relation to endothelial function have been demonstrated previously in vitro and/or ex vivo in ZDF rats. Thus, the acute and chronic treatments with CBD enhanced endothelium-dependent vasorelaxation in mesenteric arteries from ZDF rats (but not their controls) [20,21]. The above observations show that the beneficial vascular effects of CBD on Ach-mediated relaxation may be present mainly in the case of vascular dysfunction. In contrast to the positive influence of CBD on Ach-induced relaxation, its direct vasodilatory effects in hypertensive patients were either reduced in human pulmonary arteries [10] or no changed in human mesenteric arteries [19] and in patients with type 2 diabetes in isolated pulmonary arteries [10] but blunted in mesenteric arteries [21]. In contrast to Ach, CBD diminished relaxant potency of SNP in small mesenteric G3 arteries of SHR and SHAM rats. We can only speculate that it might result from either CBD’s direct influence on smooth muscle cells or via modulation of endothelium-dependent SNP-mediated effects. Thus, SNP was able to improve endothelium dependent relaxation induced by Ach independently of NO release [53] or NO dependently, as downstream effect of the calcium rise [54]. However, the detailed mechanism of this phenomenon needs further research.

The mechanism(s) involved in the beneficial influences of CBD on hypertension depend(s) on the vessel diameter. They may be partly associated with its NO-dependent or both NO- and COX-dependent activating effects in aortas and mesenteric G3 arteries, respectively. The CBD-induced augmentation of the vasorelaxant response to Ach was inhibited by L-NAME or L-NAME and indomethacin in the aortas of SHR and the mesenteric G3 arteries of both DOCA-salt rats and SHR, respectively. The potential involvement of both of the above mechanisms is also suggested by the CBD-stimulated increases in *NOS3* gene expression in all the vessels (the only exception was the aortas of DOCA-salt rats) and *PGIS* gene expression in mesenteric G3 arteries of DOCA-salt rats and SHR. eNOS was upregulated in the aortas of SHR only. By contrast, *PGIS* was downregulated in the aortas of both hypertensive models, but the COX-1 protein was upregulated in the aortas of SHR. Our results are in agreement with previous findings on the mesenteric arteries of ZDF rats, in which COX-mediated and/or NO-mediated mechanisms were responsible for the amplificatory influences of CBD on the responses to Ach [21].

Moreover, we determined that the Ach-mediated vasorelaxation in CBD-treated hypertensive rats was mainly NO-dependent in the aortas of both hypertensive models and in the mesenteric G3 arteries of DOCA-salt rats, whereas it was dependent on both NO and COX pathways in SHR. It has been previously shown that CBD increases the phosphorylation of eNOS in human aortic endothelial cells, or that its vasodilatory effect is inhibited by L-NAME, suggesting that the production of NO at least partially underlies the endothelium-dependent vasorelaxant effect of CBD in human mesenteric [19] and pulmonary arteries [10].

We found that the expression of *KCNN4* in aortas and mesenteric arteries and *KCNN3* in mesenteric G3 arteries increased in CBD-treated hypertensive rats. Therefore, EDH, which is related to the activation of these two K_Ca_, could also be responsible for the beneficial influence of CBD in maintaining endothelium-dependent vasorelaxation in arteries when either vasodilator NO or prostanoids become less effective [21].

The third beneficial vascular effect of CBD in hypertension is its influence on the vascular endocannabinoid system. Firstly, chronic CBD treatment caused increased expression of *Cnr1* and *Cnr2* in both the vessels of DOCA-salt rats and SHR, with the exception of CB_1_ receptors, whose mRNA and protein expression decreased in the mesenteric G3 arteries of DOCA-salt rats. We described, in the previous subsection, the potential protective role of vascular CB_1_ receptors [4,9,11]. Moreover, we cannot exclude that CB_2_ receptors also had beneficial effects in this study, as they are associated with anti-inflammatory actions and are involved in the CBD-mediated improvement of endothelium-dependent vasorelaxation in ZDF rats [20]. Secondly, CBD increased the aorta levels of certain endocannabinoids known for their vasorelaxant properties, i.e., AEA, 2-AG and PEA, in DOCA-salt rats, and tended to not to affect the hypertension-increased levels of OEA and NAGly in SHR. It seems that CBD induces changes mainly opposite to those evoked by hypertension. However, it caused increases in endocannabinoid levels mainly in DOCA-salt rats, for which we did not observe changes in hypertension. According to the rule, it decreased the levels of NAGly in DOCA-salt rats and AEA in SHR, which were enhanced by hypertension. Similarly, CBD induced the augmentation of vasorelaxing endocannabinoids in the lungs of experimental pulmonary hypertensive rats that were not modified by hypertension [12] and decreased the cardiac levels of 2-AG and OEA and plasma levels of AEA and LEA, which were increased in SHR [13]. Remiszewski et al. demonstrated that two weeks of CBD administration at 10 mg/kg counteracted pro-oxidant effects in the hearts and plasma of DOCA-salt rats and SHR. Thus, we cannot exclude the idea that the antioxidant properties of CBD additionally intensify its beneficial vascular effects.

In summary, in our study, CBD improved the endothelial function in hypertension, but it failed to affect BP. It should be kept in mind that the normalization of endothelial function does not necessarily affect BP [3]. For example, statins, with their confirmed protective role in the cardiovascular system, attenuate BP in several hypertensive animal models [55] but not in others (including SHR) [56,57]. Similarly, the endothelium-specific deletion of the mineralocorticoid receptor improved endothelial function and attenuated vascular inflammation in DOCA-salt-induced vascular dysfunction in mice, but it did not reduce BP [58].

CBD seems to be a safe and well-tolerated drug in the context of its vascular effects [5]. We determined its vasoprotective properties in normotensive controls as well—i.e., it reduced the width of media and enhanced the Ach-induced endothelium vasorelaxation in the mesenteric G3 arteries of WKY rats, despite the downregulation of eNOS, *KCNN4* and *KCNN3*. In SHAM rats, the effect was less visible, at least partly due to elevated eNOS expression but also reduced PGIS, *KCNN4* and *KCNN3* expression. The only adverse vascular effect was the attenuation of endothelium-independent relaxation in the mesenteric G3 arteries of SHAM rats. Some unwanted cardiac effects of chronic CBD administration in normotensive controls observed in our previous studies were probably associated with cardiac and plasma lipid peroxidation [13,29].

### 3.3. Limitations

We did not notice a hypotensive effect of CBD, despite its vasoprotective properties. Therefore, it would be interesting to use higher doses of CBD and/or extend the time of its administration or apply another hypertension model, including renovascular hypertension [32]. Moreover, since sexual dimorphism has been observed in CBD-based therapy against pain [59], since it improved mice’s memory behavior in a sex-specific manner [60] and since sexual dimorphism was observed in the vasodilatory effects of cannabinoids in mesenteric arteries isolated from WKY and SHR animals [61], the question arises as to whether CBD would be an effective hypotensive agent in female hypertensive rats. Both European [62] and North American [63] hypertension guidelines promote, alongside lifestyle changes, the use of combination therapies. Therefore, it would be interesting to compare standard guideline-based therapy to CBD-based endothelial-function-directed add-on therapy, in order to determine the overall preclinical and clinical efficacy of an endothelium-targeted approach to potentially prevent or even limit the endothelial dysfunction and remodeling of vessels. It is important to mention that polypharmacological complexity of CBD that offers therapeutic potential might also be also responsible for occurrence of variety of adverse effects including drug-drug [7,64], drug-food [65] and drug-comorbidities [10] interaction. Thus, regarding the potential negative effects of CBD and its increasing availability, rigorous clinical trials although challenging, are required to verify the preclinical data.

## 4. Materials and Methods

### 4.1. Animals

All the animal care, surgical procedures and experimental protocols were conducted in accordance with European Directives (2010/63/EU) and Polish legislation and were approved by the Local Animal Ethics Committee in Olsztyn (Poland, project code: 80/2017, approved 28 November 2017). The animal studies are reported to be in compliance with the ARRIVE guidelines [66]. The study was performed following the principles of replacement, refinement or reduction (the 3Rs).

Male Wistar, spontaneously hypertensive rats (SHR) and Wistar–Kyoto (WKY) rats were obtained from the Center for Experimental Medicine of the Medical University of Białystok. (Poland). The rats were housed at constant humidity (60 ± 5%) and temperature (22 ± 1 °C) and were kept under a 12/12 h light/dark cycle. Animals had free access to standard pelleted rat chow and tap water ad libitum unless otherwise noted.

DOCA-salt hypertension was induced in male Wistar rats (5–6 weeks old; initially weighing 200–300 g) as described previously [13,29]. All the animals were anaesthetized with an intraperitoneal (i.p.) injection of pentobarbital sodium (70 mg/kg, i.e., ~300 μmol/kg, Biowet, Puławy, Poland) and underwent unilateral nephrectomy. After seven days of recovery, the rats were divided into two groups: (1) hypertensive animals (DOCA-salt), in which hypertension was induced by subcutaneous injections of 11-deoxycorticosterone acetate (DOCA 25 mg/kg, i.e., ~67 μmol/kg; 0.4 mL/kg, Sigma-Aldrich, Munich, Germany) twice weekly for 4 weeks and drinking water was replaced with 1% NaCl solution, and (2) normotensive animals (SHAM), which received the vehicle for DOCA (*N*,*N*-dimethylformamide, Sigma-Aldrich) twice weekly and drank tap water.

Spontaneously hypertensive rats: Male 8–9-week-old SHR weighing 250–350 g and age-matched normotensive WKY rats weighing 290–380 g were used in the experiments.

### 4.2. Chronic Treatment with Cannabidiol

Hypertensive and normotensive rats were injected intraperitoneally (i.p.) with (−)-cannabidiol (CBD; THC Pharm GmbH, Frankfurt, Germany) or its vehicle (ethanol, Tween 80 (Sigma-Aldrich), 0.9% NaCl—3:1:16) every 24 h for 14 days in the following 4 groups: hypertensive (1) DOCA-salt and (2) SHR, and their normotensive controls, (3) SHAM and (4) WKY rats, as described previously [13,29].

Systolic blood pressure (SBP) was measure in conscious rats using the non-invasive tail-cuff method (ADInstruments, Sydney, Australia) before the first CBD dose or its vehicle and twenty-four hours after the final dose of CBD. Rats with SBP equal to or higher than 150 mmHg were regarded hypertensive and underwent an organ bath or a myography procedure and biochemical and histochemical evaluations.

### 4.3. Vessel Preparation

Twenty-four hours after the last dose of CBD or its vehicle, rats were anaesthetized with pentobarbitone sodium (70 mg/kg, i.e., 300 µmol/kg i.p.). The vessel preparation and experimental procedure have been described in detail previously [4,10]. Following sacrifice, the aorta and mesenteric arterial bed were dissected rapidly and placed into a cold Krebs–Henseleit buffer with the following composition (in mM): NaCl 118; KCl 4.8; CaCl_2_ 2.5; MgSO_4_ 1.2; NaHCO_3_ 24; KH_2_PO_4_ 1.2; glucose 11; and EDTA 0.03 at pH 7.4. The thoracic aortas (3–5 mm length ring) and the third order of the superior artery (G3; 2 mm length segments) were carefully cleaned to remove adherent tissue. The aortas were suspended on stainless-steel wires in 10 mL organ chambers. The isometric muscle tension was recorded using a force displacement transducer (PIM 100RE, BIO-SYS-TECH, Białystok, Poland) and displayed on a computer. Mesenteric G3 segments (~250 µm in internal diameter) were mounted in a Mulvany-Halpern-type wire myograph (Model 620 M, Danish Myo Technology, Aaerhus, Denmark). The isometric muscle tension was measured and recorded on the LabChart 7.3.7 Pro (ADInstruments, Hastings, UK). All the vessels were kept at 37 °C aerated with 95% O_2_ and 5% CO_2_ Krebs–Henseleit buffer and were allowed to equilibrate: 60 min (resting tension: ~14.7 mN) for thoracic aortas and 45 min (resting tension: ~2.5 mN) for mesenteric G3 arteries.

### 4.4. Concentration-Response Curves

After an equilibration period, to check arteries viability each vessel was pre-contracted with 120 mM KCl followed by thorough washout. Next the endothelial integrity was assessed by pre-constricting rings submaximally with the α_1_-adrenoceptor agonist (−)-phenylephrine (aortas: hypertensive rats, 0.03 μM; normotensive rats, 0.3 μM; and mesenteric G3 arteries, 3–10 μM), followed by relaxation with acetylcholine (Ach), 1 or 10 µM, respectively. A relaxing effect of at least 90% to Ach was considered an endothelium-intact vessel. After washout, concentration response curves (CRCs) were determined by the cumulative addition of increasing concentrations of appropriate agonists (in each individual preparation, only one experimental curve was constructed).

To examine the vascular relaxant function, endothelium-intact aortas and mesenteric G3 arteries were pre-constricted submaximally with phenylephrine: aortas of hypertensive rats, 0.03 μM; aortas of normotensive rats, 0.3 μM; and mesenteric G3 arteries, 3–10 µM. Then, rings from each group were exposed to Ach (aortas and mesenteric G3 arteries: 0.001–30 µM) or SNP (aortas and mesenteric G3 arteries: 0.0001–30 µM). All the experiments were performed in paired arteries—the solvent-control responses were compared with the drug-treated-group responses on arteries from the same animal.

To test the involvement of nitric oxide or cyclooxygenase (COX)-dependent pathways in Ach-mediated vasorelaxation in aortas and/or mesenteric G3 arteries, rings were pre-treated with the eNOS or COX inhibitor L-NAME (300 µM [10]) or indomethacin (INDO, 10 µM [10]) for 45 min; these were present throughout the experiment. In control tissues, the respective solvent was used instead. Each vessel was again contracted with 120 mM KCl at the end of the CRCs to determine the vessel viability.

### 4.5. Thickness of Media in Aortas and Mesenteric G3 Arteries

Thoracic aortas and mesenteric G3 arteries were fixed in 10% buffered formalin and embedded in paraffin in a routine manner. The paraffin blocks were cut into sections of 4 μm in thickness (using a model 2025 rotating microtome, Leica, Wetzlar, Germany), stained with hematoxylin and eosin (H + E) for general histological examination, and processed by immunohistochemistry to detect vWF, eNOS, CB_1_, CB_2_ and TRPV1. The quantitative analysis of thickness of media in aortas and mesenteric G3 arteries were determined with a BX41 light microscope (Olympus, Tokyo, Japan) and a video circuit and a Pentium 120 PC computer running the NIS Elements BR software for microscope image analysis. The measurement of the thickness of the middle layer of the examined blood vessel wall was performed at a uniform magnification of 200 (×10 objective and ×20 eyepiece). Representative micrographs were selected from *n* = 5 images.

### 4.6. Immunohistochemistry

To localize vWF, eNOS, CB_1_ and TRPV1 the EnVision method was used according to Baranowska-Kuczko et al. [4,10] using a polyclonal rabbit Anti-Human antibody against Von Willebrand Factor (vWF) (A 0082; DakoCytomation, Glostrup, Denmark)–1:2000; anti-eNOS antibody (ab76198, Abcam, Cambridge, UK)—1:800;) and rabbit polyclonal antibody against the CB_1_ receptor (ab23703, Abcam)—1:1000 and TRPV1 polyclonal antibody (bs-1931R, Bioss Antibodies, Woburn, MA, USA)—1:1000.

Each antibody stage had a negative control, in which the antibody was replaced with normal rabbit serum (Vector Laboratories, Burlingame, CA, USA) at the respective dilution (no staining), and a positive control. The tissue recommended by the antibody manufacturer was used in the staining: eNOS—human normal placenta tissue; CB_1_ and TRPV1—rat hippocampus; vWF and CB_2_—human tonsil tissue. All the control reactions performed gave positive results. The histological preparations were evaluated using a BX43 light microscope equipped with an Olympus DP12 digital camera under a magnification of 200× (20× lens and 10× eyepiece) and documented. In each blood vessel the percentage area stained was measured using the ImageJ software [67], and an average was calculated from the values obtained from the five vessels per case. To calculate the percentage area of staining in the vessel wall, ImageJ software version 1.53c (Java 1.8.0_172) (NIH, Bethesda, MD, USA) was used to draw around the artery, and the area stained was divided by the total area to give a percentage staining. If a transverse section of a blood vessel was captured, the lumen area was excluded from the total area before dividing by the area stained. Then, the data are expressed as the fold changes compared to the respective control groups.

### 4.7. Western Blots

The total expression levels of selected proteins were determined using a routine Western blotting procedure, which was described previously [4,10]. Briefly, samples of aortas and mesenteric G3 arteries were harvested and then lysed and homogenized in a cold radioimmunoprecipitation assay (RIPA) buffer containing a cocktail of protease and phosphatase inhibitors (Roche Diagnostics GmbH, Mannheim, Germany). The total protein concentration was measured using the bicinchoninic acid method (BCA) with bovine serum albumin (BSA) as a standard. Then, homogenates were reconstituted in Laemmli buffer, and the same amounts of protein (20 µg) were loaded onto Criterion^TM^ TGX Stain-Free Precast Gels (Bio-Rad, Hercules, CA, USA). The homogenates were separated by 10% sodium dodecyl sulfate–polyacrylamide gel electrophoresis, transferred onto nitrocellulose membranes and blocked in Tris-buffered saline with Tween-20 (TBST) and 5% non-fat dry milk or BSA. Equal protein loading was checked by Ponceau S staining. Then, the membranes were incubated overnight at 4 °C with corresponding primary antibodies at appropriate dilutions, i.e., CB_1_ (1:500; ab23703, Abcam), CB_2_ (1:500; ab3561, Abcam), TRPV1 (1:500; sc-398417, Santa Cruz Biotechnology, Santa Cruz, CA, USA), COX-1 (2 µg/mL; ab695, Abcam), COX-2 (1:500; sc-376861, Santa Cruz Biotechnology) and eNOS (1:500; ab76198, Abcam). Thereafter, to detect the proteins, the nitrocellulose membranes were incubated with the corresponding secondary antibodies conjugated with horseradish peroxidase (1:3000; Santa Cruz Biotechnology). A suitable substrate for horseradish peroxidase (Clarity Western ECL Substrate; Bio-Rad) was added, and the protein bands were quantified densitometrically using a ChemiDoc visualization system (Image Laboratory Software Version 6.0.1; Bio-Rad, Warsaw, Poland). The expression of selected proteins was quantified using stain-free gels and the total protein normalization method (Bio-Rad). All the data are expressed as the fold changes compared to the respective control groups based on five or six independent determinations.

### 4.8. RT-qPCR

Rat aortas and mesenteric G3 arteries were isolated from hypertensive SHR and DOCA-salt and normotensive WKY and SHAM animals, and further subjected to RNA isolation and gene expression analysis according to previously described methodology [10]. Briefly, total RNA was purified from up to 30 mg of tissue using a NucleoSpin TriPrep, Mini Kit for RNA, DNA, and Protein Purification (Macherey-Nagel GmbH and Co., Düren, Germany) according to the manufacturer’s protocol. The synthesis of the cDNA was performed using the iScript cDNA Synthesis Kit (Bio-Rad) following the manufacturer’s instructions. In brief, 500 ng of purified total RNA was used in a 20 μL reaction mixture containing random octamers, oligo dT-16 primers, dNTPs and RNase H+ MMLV reverse transcriptase. cDNA (2 μL) served as a template for the real-time qPCRs. The amplification of the product was performed using SsoAdvanced Universal SYBR Green Supermix (Bio-Rad). The sequences of the PCR primers for *NOS3*, prostacyclin I2 synthase (*PGIS*), *KCNN4* and *KCNN3* were previously described by Kloza et al. [40]. A set of predesigned primer pairs for *Cnr1* and *Cnr2* was purchased from Bio-Rad (PrimePCR PreAmp), as described in our previous work [10]. As an internal control, two reference genes, *GAPDH* and *cyclophilin A* [10], were tested, and *GAPDH* was chosen for further analysis. The following reaction parameters were applied: initial denaturation at 95 °C for 3 min, followed by 40 cycles of 95 °C for 1 min, 57 °C for 30 s and 72 °C for 45 s. The CFX Connect Real-Time PCR System (Bio-Rad) was used to perform the real-time quantitative PCR (RT-qPCR) assay. The reactions were run in triplicate, and the expression was analyzed using the relative quantification method modified by Pfaffl [68].

### 4.9. Determination of Endocannabinoids

Aortas and mesenteric G3 arteries were snap-frozen and kept at −80 °C. Next, the samples were pulverized in liquid nitrogen to examine the levels of endocannabinoids. *N*-arachidonoylethanolamine (AEA), 2-arachidonoylglycerol (2-AG), arachidonoylglycine (NAGly), palmitoylethanolamide (PEA), oleoylethanolamide (OEA), linolenoylethanolamide (LEA), stearoyl ethanolamide (SEA), homo-linolenyl ethanolamide (HEA), palmitoleoylethanolamide (POEA), docosahexaenoyl ethanolamide (DHEA), docosatetraenoyl ethanolamide (DEA), 2-linoleoylglycerol (2-LG) and eicosapentaenoylethanolamide (EPEA) using ultra-high-performance liquid chromatography tandem mass spectrometry (LCMS 8060, Shimadzu, Kyoto, Japan) via the Luque–Córdoba method [69]. Endocannabinoids were separated on an Agilent Poroshell 120 EC-C18 analytical column (3.0 × 150 mm, 2.7 µm particle size; Agilent, Santa Clara, CA, USA). The mobile phase consisted of 0.1% formic acid in water (A) and 0.1% formic acid in acetonitrile (B). The initial chromatographic conditions were 70% acetonitrile (ACN) in water containing 0.1% (*v/v*) formic acid as an ionizing agent. After isocratic development for 1 min, the gradient was applied: 70% B to 80% B for 5 min, 80% B to 88% B until minute 15, 88% B to 100% B until minute 16, holding at 100% B from minute 16 to minute 20, 100% B to 70% B until minute 21 and maintenance at 70% B to re-equilibrate the column until the end of the run time (another 4 min). The temperature of the chromatographic column and the flow rate were maintained at 18 °C and 800 µL/min, respectively. Octadeuterated endocannabinoids, AEA-d8, 2-AG-d8 and OEA-d4, as internal standards, were added into the samples, and all the endocannabinoids were extracted using solid-phase extraction (SPE) and analyzed in positive-ion mode (MRM). The transitions of the precursors to the product ions were as follows: *m/z* 348.3→62.15 for AEA, *m/z* 379.3→287.25 for 2-AG, *m/z* 362.10→287.25 for NAGLy, *m/z* 300.3→62.0 for PEA, 326.3→62.0 for OEA, *m/z* 324.3→62.0 for LEA, *m/z* 328.3→62.0 for SEA, *m/z* 314.5→62.0 for HEA, *m/z* 298.3→62.0 for POEA, *m/z* 372.3→62.0 for DHEA, *m/z* 376.3→62.0 for DEA, *m/z* 355.0→263.0 for 2-LG, *m/z* 346.3→62.0 for EPEA, *m/z* 356.2→63.05 for AEA-d8, *m/z* 387.3→294.0 for 2-AG-d8 and *m/z* 330.20→66.15 for OEA-d4.

### 4.10. Drugs

*N*,*N*-Dimethylformamide (DMF) and Tween-80 (Sigma-Aldrich); (−)-cannabidiol (CBD; THC Pharm GmbH, Frankfurt, Germany), anandamide-d8 (AEA-d8); 2-arachidonoyl glycerol-d8 (2-AG-d8); OEA-d4 (Cayman Chemical Company, Ann Arbor, MI, USA); pentobarbitone sodium (Biowet, Puławy, Poland); and sodium chloride (NaCl) (Chempur, Piekary Śląskie, Poland). Acetylcholine chloride, phenylephrine sodium nitroprusside and L-NAME (Sigma, Munich, Germany) were dissolved in deionized water. Indomethacin (Sigma) was dissolved in 0.5 mol/L NaHCO_3_. A stock solution (10 µM) of U46619 (9,11-Methanoepoxy PGH_2_) (Tocris, Bristol, UK) was prepared in ethanol (0.1% *v*/*v*) and diluted with deionized water, which adjusted the final concentrations of ethanol to less than 0.01% *v*/*v*. The antibodies used in the Western blots and immunohistochemistry were purchased from Abcam (CB_1_, CB_2_, COX-1, COX-2 and eNOS), Santa-Cruz Biotechnology (TRPV1, COX-2, anti-rabbit IgG horseradish peroxidase-conjugated secondary antibody), Dako (vWF) and Bioss Antibodies (TRPV1). The reagents for routine histological H + E staining and secondary antibody En Vision + Kit HRP Rabbit were obtained from Dako Denmark A/S, and normal rabbit serum was obtained from Vector Laboratories.

### 4.11. Statistical Analysis

For the relaxant responses to Ach, the SNP data are shown as percentages of relaxation compared to the contraction induced by phenylephrine. GraphPad Prism 5.0 (GraphPad Software Inc., La Jolla, CA, USA) was used to plot the mean data as sigmoidal concentration-response curves. The curves were used to determine the potency (the pEC_50_: the negative logarithm of the concentration causing the half-maximum effect) and maximal response (R_max_) values. Similarly to our previous study [10], if the CRC did not reach a clear top plateau, it was quantified as a rough measure of the maximum extent of relaxation obtainable (R_max_). The rightward shifts of CRCs relative to the control curve were calculated on the basis of the EC_50_ values. If R_max_ was attenuated by more than 50%, the pEC_50_ was not calculated. All the results are expressed as the means ± SEMs of *n* animals. All data was assessed for Gaussian distribution prior to statistical analysis and due to passing a normality test, it was analysed using parametric tests. Intergroup statistical comparisons were made by analysis of variance (ANOVA) followed by Bonferroni’s multiple comparison tests for the entire dataset. Post hoc tests were performed only if F achieved the necessary level for statistical significance and there was no significant variance inhomogeneity. Student’s *t*-tests for unpaired data were used as appropriate. Differences were considered to be statistically significant at *p* < 0.05.

## 5. Conclusions

Our work is the first to show that CBD treatment has unique endothelial (vascular)-structure- and function-protective properties in hypertensive rats. We revealed that chronic treatment with CBD reduced hypertrophy and improved the endothelium-dependent vasodilation in a vessel-size- and hypertension-model-independent manner. The mechanism by which CBD improves endothelial function in hypertension depends on the vessel diameter and may be partly associated with its NO-; K_Ca_3.1-; or NO, COX(PGI_2_) and EDH (K_Ca_3.1 and K_Ca_2.3)-dependent effect on aortas and mesenteric G3 arteries, respectively. Additionally, the endocannabinoid system might play a beneficial role in hypertension because of the hypertension-model- dependent enhancements in the levels of certain vasodilatory endocannabinoids and in the expression levels of CB_1_ and/or CB_2_ receptors in the vasculature of hypertensive subjects. Importantly, CBD showed vasoprotective properties in normotensive rats, and we did not observe any vascular unwanted effects of CBD in hypertensive or normotensive control rats.

Improvements in endothelial (vascular) function are important in the prevention and therapy of essential hypertension. Targeting the hypertension-associated adverse vascular changes by using CBD as an add-on therapy might represent a promising therapeutic strategy for patients with endothelial-dysfunction–associated diseases, in addition to achieving absolute reductions in BP. Moreover, the vasoprotective and anti-inflammatory properties of CBD required further research to establish whether these beneficial vascular effects can be transferred to the human cardiovascular system and the treatment of cardiovascular disorders.

## Figures and Tables

**Table 1:** The influence of cannabidiol (CBD) and its vehicle on the vasorelaxant effects of acetylcholine (Ach), also in the presence of L-NAME (300 µM) and indomethacin (INDO, 10 µM), and sodium nitroprusside (SNP) in the endothelium-intact aortas isolated from deoxycorticosterone-salt (DOCA-salt) and spontaneously (SHR) hypertensive rats and their respective normotensive controls, sham-operated (SHAM) and Wistar-Kyoto (WKY) rats, respectivey.

Group AORTA	SHAM	SHAM + CBD	DOCA-Salt	DOCA-Salt + CBD	WKY	WKY + CBD	SHR	SHR + CBD
Ach	(8)	(8)	(6)	(7)	(8)	(8)	(8)	(8)
pEC_50_	6.6 ± 0.1	6.5 ± 0.1	6.5 ± 0.1	6.9 ± 0.1 ^∆^	6.9 ± 0.1	7.2 ± 0.1	7.1 ± 0.1	7.5 ± 0.1 ^∆^
R_max_ (%)	92.9 ± 2.8	93.4 ± 4.6	77.3 ± 2.4	91.2 ± 5.8	98.5 ± 7.2	104.0 ± 7.9	83.7 ± 9.4	106.8 ± 5.0
Ach + L-NAME	(4)	(4)	(4)	(4)	(5)	(5)	(6)	(6)
pEC_50_	-	-	-	-	-	-	-	-
R_max_ (%)	0.5 ± 3.1 ^###^	25.5 ± 2.1 ^###^	23.7 ± 6.7 ^###^	37.9 ± 21.1 ^##^	17.8 ± 1.5 ^###^	7.0 ± 10.8 ^###^	9.3 ± 7.2 ^###^	9.5 ± 13.7 ^###^
Ach + INDO	(6)	(4)	(4)	(4)	(5)	(5)	(6)	(6)
pEC_50_	6.3 ± 0.1	6.9 ± 0.1 ^#,a^	6.5 ± 0.1	7.3 ± 0.1 ^#,a^	6.9 ± 0.1	6.8 ± 0.1 ^#,a^	7.1 ± 0.1	7.4 ± 0.1
R_max_ (%)	76.3 ± 4.9 ^##^	101.6 ± 8.1	83.8 ± 8.4	81.3 ± 5.0	97.9 ± 11.1	101.2 ± 6.9	91.3 ± 9.1	95.9 ± 7.0
SNP	(7)	(7)	(7)	(7)	(8)	(6)	(7)	(8)
pEC_50_	8.4 ± 0.1	8.5 ± 0.1	7.9 ± 0.1 **	7.7 ± 0.1	7.6 ± 0.1	7.9 ± 0.1	7.6 ± 0.1	7.6 ± 0.1
R_max_ (%)	107.3 ± 1.8	107.2 ± 1.2	102.6 ± 1.1	110.3 ± 3.6	106.3 ± 6.0	121.5 ± 9.1	115.7 ± 6.3	106.3 ± 6.0

Values are based on the concentration-response curves shown in Figure 1, Figure 2 and Figure 3. CBD 10 mg/kg or its vehicle were injected i.p. every 24 h for 14 days. Aortas were isolated 24 h after the final dose of CBD or its vehicle. The numbers of animals are shown within parentheses. Vasodilator responses are shown as a percentage of the isometric contraction induced by phenylephrine (hypertensive 0.03 μM, normotensive rats 0.3 μM). ^∆,#^ *p* < 0.05; **^,##^ *p* < 0.01; ^###^ *p* < 0.001 compared to the * SHAM/WKY, ^∆^ DOCA-salt/SHR and ^#^ Ach, as determined by one-way ANOVA followed by Bonnferoni’s multiple comparison test or ^a^ Student’s *t*-test for unpaired data.

**Table 2:** The influence of cannabidiol (CBD) and its vehicle on the vasorelaxant effects of acetylcholine (Ach), also in the presence of L-NAME (300 µM) and indomethacin (INDO, 10 µM), and sodium nitroprusside (SNP) in the endothelium-intact small mesenteric (G3) arteries isolated from deoxycorticosterone-salt (DOCA-salt) and spontaneously (SHR) hypertensive rats and their respective normotensive controls, sham-operated (SHAM) and Wistar-Kyoto (WKY) rats, respectively.

Group G3	SHAM	SHAM + CBD	DOCA-Salt	DOCA-Salt + CBD	WKY	WKY + CBD	SHR	SHR + CBD
Ach	(7)	(5)	(6)	(6)	(8)	(8)	(7)	(8)
pEC _50_	6.5 ± 0.1	6.8 ± 0.1	6.4 ± 0.1	6.9 ± 0.1 ^∆∆^	6.5 ± 0.1	6.9 ± 0.1 *	6.5 ± 0.1	7.9 ± 0.1 ^∆∆∆^
R_max_ (%)	95.9 ± 2.7	97.7 ± 2.6	95.7 ± 3.2	96.3 ± 2.1	81.6 ± 7.3	94.3 ± 2.0	78.0 ± 6.5	87.3 ± 6.6
Ach + L-NAME	(4)	(4)	(4)	(6)	(6)	(6)	(5)	(5)
pEC_50_	6.1 ± 0.1 ^#^	-	6.8 ± 0.1 ^#^	6.3 ± 0.1 ^##^	6.1 ± 0.1 ^#^	6.0 ± 0.1 ^###^	-	-
R_max_ (%)	61.7 ± 20.9 ^#^	31.3 ± 18.5 ^##^	78.5 ± 7.4	64.4 ± 8.4 ^##^	50.3 ± 8.2	71.0 ± 6.9 ^##^	49.6 ± 3.9 ^##^	28.4 ± 14.0 ^###^
Ach + INDO	(5)	(5)	(5)	(6)	(5)	(6)	(5)	(5)
pEC_50_	6.6 ± 0.1	6.1 ± 0.1 ^##,a^	6.6 ± 0.1	6.5 ± 0.1 ^#^	6.5 ± 0.1	6.8 ± 0.1	6.3 ± 0.1	6.0 ± 0.1 ^###,a^
R_max_ (%)	79.9 ± 4.8	71.3 ± 10.0	88.7 ± 10.4	91.4 ± 1.6	70.3 ± 16.3	62.9 ± 5.6 ^###^	76.6 ± 1.6	51.8 ± 1.0 ^#^
SNP	(7)	(6)	(5)	(5)	(5)	(5)	(6)	(7)
pEC _50_	6.8 ± 0.2	5.8 ± 0.1 ***	6.0 ± 0.1 **	6.3 ± 0.1	6.8 ± 0.1	6.4 ± 0.1	6.8 ± 0.1	6.0 ± 0.1 ^∆∆∆^
R_max_ (%)	65.1 ± 6.4	57.7 ± 8.0	57.2 ± 6.1	53.0 ± 8.5	71.2 ± 8.4	90.0 ± 7.8	61.0 ± 9.5	71.1 ± 7.0

Values are based on the concentration-response curves shown in Figure 1, Figure 2 and Figure 3. CBD 10 mg/kg or its vehicle were injected i.p. every 24 h for 14 days. Small mesenteric (G3) arteries were isolated 24 h after the final dose of CBD or its vehicle. The numbers of animals are shown within parentheses. Vasodilator responses are shown as a percentage of the isometric contraction induced by phenylephrine (3–10 μM). *^,#,∆^ *p* < 0.05; **^,^^∆∆,^^##^ *p* < 0.01; ***^,^^∆∆∆,^^###^ *p* < 0.001 compared to the * SHAM/WKY, ^∆^ DOCA-salt/SHR and ^#^ Ach, as determined by one-way ANOVA followed by Bonnferoni’s multiple comparison test or ^a^ Student’s *t*-test for unpaired data.

## Data Availability

The data presented in this study are available on request from the corresponding author. The data are not publicly available due to privacy.

## Abbreviations

Ach	Acetylcholine
2-AG	2-Arachidonoylglycerol
AEA	Anandamide
ANOVA	Analysis of variance
BP	Blood pressure
*Cnr1*	Cannabinoid receptor 1 protein coding gene
*Cnr2*	Cannabinoid receptor 2 protein coding gene
COX	Cyclooxygenase
CRCs	Concentration response curves
CBD	Cannabidiol
DMF	*N*,*N*-Dimethylformamide
DEA	Docosatetraenoyl ethanolamide
DHEA	Docosahexaenoyl ethanolamide
eNOS	Endothelial nitric oxide synthase
EPEA	Eicosapentaenoyl ethanolamide
FAAH	Fatty acid amide hydrolase
HEA	Homo-γ-linolenyl ethanolamide
HR	Heart rate
LEA	Linolenoyl ethanolamide
2-LG	Linoleoylglycerol
i.p.	Intraperitoneally
K_Ca_3.1	Intermediate potassium calcium-activated channel
K_Ca_2.3	Small potassium calcium-activated channel
*KCNN3*	Potassium calcium-activated channel subfamily N member 3 protein coding gene
*KCNN4*	Potassium calcium-activated channel subfamily N member 4 protein coding gene
kDA	Kilodaltons
LC-MS	Ultrahigh performance liquid chromatography-tandem mass spectrometry
L-NAME	N^G^-Nitro-L-arginine methyl ester
MRM	Multiple reaction monitoring
NAGly	*N*-Arachidonoyl glycine
N.D.	Not determined
NO	Nitric oxide
OEA	Oleoyl ethanolamide
PEA	Palmitoyl ethanolamide
POEA	Palmitoleoyl ethanolamide
*PGIS*	Prostaglandin I2 synthase protein coding gene
Phe	Phenylephrine
PPAR	Peroxisome proliferator-activated receptors
RIPA	Radioimmunoprecipitation assay
RT-PCR	Real time reverse transcription-polymerase chain reaction
SBP	Systolic blood pressure
SHAM	Sham operated Wistar rats
SHR	Spontaneously hypertensive rats
SNP	Sodium nitroprusside
SOD	Superoxide dismutase
SEA	Stearoyl ethanolamide
TRPV1	Transient receptor potential vanilloid type 1
vWF	Von Willebrand factor
WKY	Wistar Kyoto Rats
ZDF	Zucker Diabetic Fatty rats
